# Non-Melanocytic Histopathological Clues for Melanoma Diagnosis: A Practical Review of Solar Elastosis, Stromal Regression, and Epidermal Reaction Patterns. Do Old-School Clues Still Matter?

**DOI:** 10.3390/dermatopathology13030032

**Published:** 2026-07-13

**Authors:** Michail Sofopoulos

**Affiliations:** Department of Histopathology, Andreas Syngros Hospital of Cutaneous and Venereal Diseases, 16121 Athens, Greece; msofopoulos@gmail.com

**Keywords:** epidermal effacement, melanoma, non-melanocytic histopathologic clues, pseudoepitheliomatous hyperplasia, regression, solar elastosis, tertiary lymphoid structures

## Abstract

Histopathologic melanoma diagnosis extends beyond melanocytic cytology to encompass non-melanocytic features: solar elastosis patterns, stromal regression, adnexal relationships, epidermal reaction patterns, and the host inflammatory response. These “old school” low-power clues are particularly valuable on sun-damaged skin, where benign nevi, reactive melanocytic hyperplasia, and melanoma in situ share overlapping features. Quantitative data support two elastosis-based signs: the “umbrella sign” (reduced elastosis beneath the lesion’s central third; PPV (Positive Predictive Value) for nevus, 96%, and NPV (Negative predictive Value) for melanoma, 74%; calculated from raw cohort data) and the “purple fiber sign” (100% specificity, 30% sensitivity for nevus), both from a cohort of 81 actinically damaged lesions. Regression—identified by compressed elastic layers displaced to the reticular dermis, fibrosis, melanophages, and inflammation—aids diagnosis but complicates distinction from surgical scar. The maturation state of tertiary lymphoid structures (TLSs) within the regression zone, ranging from immunosuppressive immature aggregates to anti-tumoral mature structures with germinal centers, may explain the variable prognostic significance of histologic regression. Epidermal hyperplasia over thick melanomas reflects angiogenesis-related changes, while effacement is a practical red flag in spitzoid lesions. Ancillary tests are most productive when morphology has already framed the differential. These non-melanocytic clues remain indispensable as the foundation for rational ancillary testing.

## Simple Summary

Distinguishing a harmless mole from a melanoma can be one of the most difficult judgments in skin pathology, especially in older, sun-damaged skin, where the two can look deceptively similar. Pathologists usually focus on the pigment-producing cells themselves, but the surrounding skin also carries useful information. This review brings together the clues found outside these cells—the pattern of sun-related fiber damage beneath a lesion, the reaction of the supporting tissue, signs that the body’s immune system has partly destroyed the lesion, and the behavior of nearby hair follicles and glands. We explain how much scientific evidence supports each clue, note that several are still preliminary, and offer a simple step-by-step way to combine them with modern laboratory tests. The aim is to help pathologists reach a more confident diagnosis and, ultimately, improve care for patients.

## 1. Introduction

Melanocytic cytology is not reliably binary: epithelioid, spindled, and even pleomorphic-appearing melanocytes can occur in both nevi and melanoma, so cytology alone may not determine biological behavior [1]. For this reason, pattern analysis in melanocytic pathology has long prioritized architecture plus contextual features—epidermal change, dermal stroma, adnexal involvement, solar elastosis, stromal reaction and regression, and inflammation-features often colloquially labeled “old school” [1,2]. Rather than being obsolete, several of these background features have been formalized in the literature and remain clinically practical, particularly for lesions arising on actinically damaged skin [3,4].

Non-melanocytic features can act as an external “truth test” for the lesion’s interaction with its microenvironment, which may diverge between long-standing benign proliferations and newer malignant ones [3]. A benign lesion may “shield” dermis from further actinic injury, whereas melanoma may expand over pre-existing elastotic dermis, displace elastotic bands, distort adnexa, and provoke regression-type stromal responses [3,5]. These signatures are often visible at scanning magnification and can guide deeper levels, targeted scrutiny of suspicious foci, and rational selection of ancillary tests [3]. The review is deliberately detailed so that it can serve as a practical reference; the consolidated three-step framework and summary table in Section 6 distill the core points for rapid use at the microscope.

## 2. Solar Elastosis Patterns

Solar elastosis reflects cumulative ultraviolet damage to dermal collagen and elastic fibers, producing thickened, curled, basophilic elastotic material in the papillary and upper reticular dermis [3]. In normal sun-exposed skin, thin elastic fibers in the papillary dermis are arranged perpendicular to the skin surface in a characteristic “candelabra” or “fork” pattern, while coarser, branched, wavy elastic fibers in the reticular dermis run parallel to the surface [5]. Melanocytic lesions can alter this baseline architecture in diagnostically informative ways, and recognition of these patterns requires deliberate low-power assessment with elastin histostain or Elastic Verhoeff–van Gieson (EVG) stain, often superior to routine H&E for highlighting subtle changes [3,5].

### 2.1. The Umbrella Sign

The “umbrella sign” is defined as a lower solar elastosis score beneath the central one-third of a melanocytic lesion compared with the adjacent dermis on sun-damaged skin [3]. To orient the reader, the umbrella and purple fiber signs should be regarded as promising but still preliminary observations rather than as established diagnostic criteria; their evidence level is summarized alongside the more firmly established clues in Table 1. This sign was formally studied by Wood and Harvey in a series of 81 melanocytic proliferations arising on actinically damaged skin [3]. The umbrella sign was present in 49/53 nevi (92%) but only 2/28 melanomas (7%), yielding a positive predictive value for nevus of 96% and a negative predictive value (i.e., absence favoring melanoma) of 74% [3]. It should be emphasized that these quantitative figures derive from this single cohort and are best regarded as promising but preliminary rather than as established diagnostic thresholds [3]. The qualitative concept, however, has since been independently described and endorsed as a practical diagnostic clue in a separate review, lending external support to the underlying principle even though the specific predictive values await independent validation [6]. The same underlying principle is articulated in a standard dermatopathology reference, which interprets solar elastosis beneath a melanocytic lesion as an indirect sign of a lesion that developed later in life and is therefore more likely to be melanoma, on the assumption that a long-standing nevus protects the dermis beneath it and preserves the sub-lesional collagen and elastic tissue [7]. This convergent reasoning from an independent source reinforces the biologic rationale for the sign, distinct from the original cohort’s quantitative claims [7]. A recent peer-reviewed update on lentigo maligna likewise invokes the same principle, stating that in true melanocytic nevi the melanocytes continue to protect the underlying dermis against sun damage, forming what is known as the umbrella sign, such that solar elastosis is almost never seen beneath these lesions [8].

**Figure 1:**
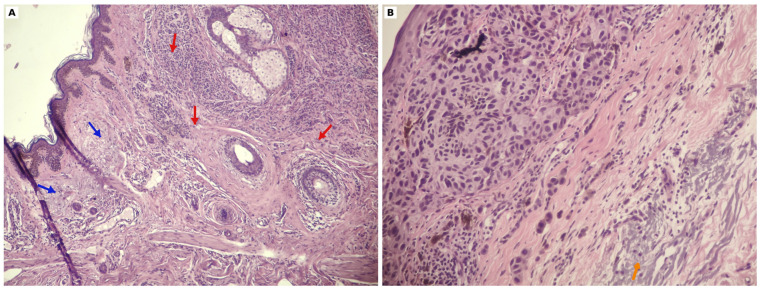
The “umbrella sign”—reduced dermal solar elastosis beneath a melanocytic proliferation. (**A**) Benign compound nevus in sun-damaged skin: the dermis directly beneath the nevus shows reduced/attenuated solar elastosis (red arrows), the lesion acting as an “umbrella” that shields the underlying dermis from cumulative ultraviolet damage; in the adjacent dermis, where no nevus is present overhead, this protective effect is absent and conspicuous basophilic solar elastosis persists (blue arrows) (H&E, ×100). (**B**) Invasive melanoma in sun-damaged skin: this shielding effect is lost, and basophilic solar elastosis persists in the dermis at the base of the lesion (orange arrow). The presence of preserved (rather than reduced) elastosis beneath an atypical melanocytic proliferation is a clue that favors melanoma over a benign nevus (H&E, ×200). This figure is an original image from the Department of Histopathology, Andreas Syngros Hospital, Athens, Greece, and illustrates previously established histopathological findings.

Mechanism and interpretation: The umbrella sign reflects a “sunscreen” effect: long-standing nevi are thought to have been present for years or decades, during which time the nevus cells and overlying epidermis physically shield the underlying papillary dermis from continued ultraviolet exposure, preventing accumulation of additional elastotic material in that zone [3]. In contrast, melanomas-generally of more recent onset-arise over pre-existing sun-damaged dermis and do not produce this protective shielding phenomenon [3]. As melanoma grows, it may expand laterally and vertically over elastotic dermis without reducing the underlying elastosis burden; in fact, invasive melanoma may displace or compress the elastotic band downward (Figure 1) [3,4].

Technical considerations: Assessment of the umbrella sign must focus on the central one-third of the lesion, not the periphery [3]. Many intradermal nevi exhibit a “shoulder phenomenon,” in which peripheral nevus cells (particularly at the lateral shoulders of a compound nevus) sit above dermis where elastosis reappears because the peripheral nevus is thinner or the shielding effect is incomplete [3]. Evaluating the periphery alone can therefore lead to a false-negative umbrella sign [3]. Additionally, very small nevi or recently developed junctional nevi may lack a well-developed umbrella sign simply because insufficient time has elapsed for the protective effect to become evident; thus, absence of the sign is not diagnostic of melanoma in isolation [3].

Elastosis scoring: Wood and Harvey used a semiquantitative 4-point scale (0 = none, 1 = low, 2 = moderate, 3 = severe; adapted from WHO Classification of Skin Tumours, 4th edition) to score elastosis density beneath the central third of the lesion and in the adjacent dermis [3]. An umbrella sign was defined as a score in the lesional dermis at least one grade lower than in the adjacent dermis [3]. In practice, even a simple visual comparison (less elastosis under the lesion centrally vs. adjacent skin) can be highly informative at the microscope [3].

Pitfalls: Thick invasive melanomas may destroy the elastotic band centrally, creating a zone of reduced elastosis that superficially resembles an umbrella sign [3]. In these cases, the periphery of the melanoma typically reveals displaced or compressed elastosis (rather than true reduction), and the overall architectural features of melanoma (asymmetry, poor circumscription, high-grade cytology, and lack of maturation) are evident [3]. Desmoplastic melanomas and desmoplastic nevi can also displace elastosis and require careful integration of all histologic features [3].

### 2.2. The Purple Fiber Sign (High Specificity Pro-Nevus)

**Figure 2:**
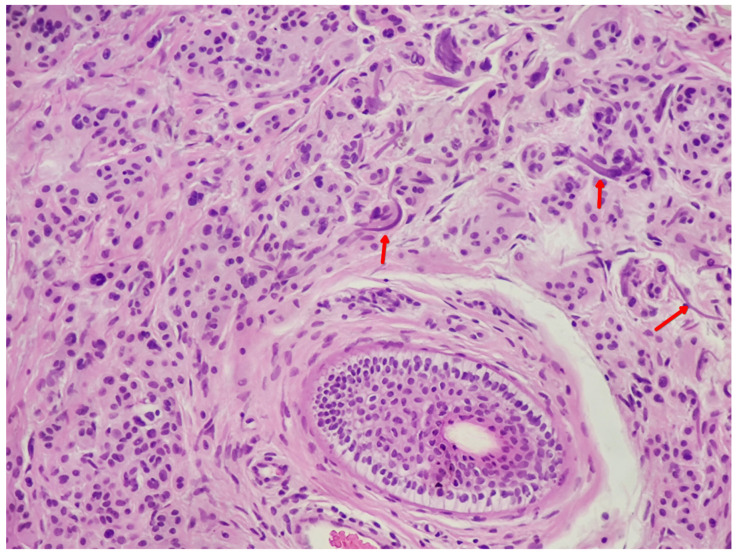
Purple fiber sign-purple-tinged elastotic fibers (arrows) within intradermal nevus component (H&E, ×200). This figure is an original image from the Department of Histopathology, Andreas Syngros Hospital, Athens, Greece, and illustrates previously established histopathological findings.

The “purple fiber sign” refers to entrapped elastotic fibers with a distinct purple (rather than the usual pink or gray-blue) tinctorial shift within the intradermal component of a melanocytic lesion, best appreciated on H&E (Figure 2) [3]. In Wood and Harvey’s cohort, purple fibers were identified in 16/53 nevi (30% of nevi, particularly intradermal nevi) and in 0/28 melanomas, conferring 100% specificity for nevus (though only 30% sensitivity) [3]. The authors noted that visibility of the purple fiber sign depends on local H&E staining protocols and pH variations, which can influence the hue of elastotic material [3]. When present, the purple fiber sign is a supportive clue for benignity and can be reassuring in an otherwise ambiguous lesion; like the umbrella sign, however, it derives from the same single cohort and should be treated as a promising but still preliminary observation pending independent validation [3].

### 2.3. Displacement/Compression of Solar Elastosis (Pro-Melanoma)

Conversely, downward displacement or compression of the solar elastosis band can support melanoma [3,4]. Horenstein et al. described depression of the dermal solar elastosis band in invasive melanoma on sun-damaged skin, consistent with expansile growth pushing elastotic dermis deeper into the reticular dermis [4]. Kamino et al. further characterized a “pushing border” phenomenon, in which the invasive melanoma and its associated stroma push the pre-existing papillary dermis elastic fibers downward, forming a compressed layer that can be highlighted with an elastin histostain [5]. This is distinct from the umbrella sign’s protective reduction in elastosis [3,5].

In thick invasive melanomas, central destruction of the elastotic band may occur, mimicking an umbrella-like clearing [3]. In these cases, careful examination of the lesion’s periphery typically reveals displaced elastosis consistent with malignant expansion rather than nevus-type shielding [3]. The overall architectural context (asymmetry, invasion depth, cytologic atypia, lack of maturation) clarifies the diagnosis [3].

## 3. Stromal Reaction and Regression

### 3.1. Regression-Type Fibrosis, Melanophages, and Vascular Change (Pro-Melanoma)

Regression in melanoma is typically accompanied by combinations of dermal fibrosis/scarring, increased melanophages, altered vessels (often ectasia/telangiectasia), and inflammation [5,9]. When a relatively subtle junctional proliferation sits above a conspicuously activated dermis with patchy fibrosis and melanophages, the microenvironment may be signaling prior tumor presence and immune-mediated regression-supporting melanoma in situ or superficially invasive melanoma [5,9].

**Figure 3:**
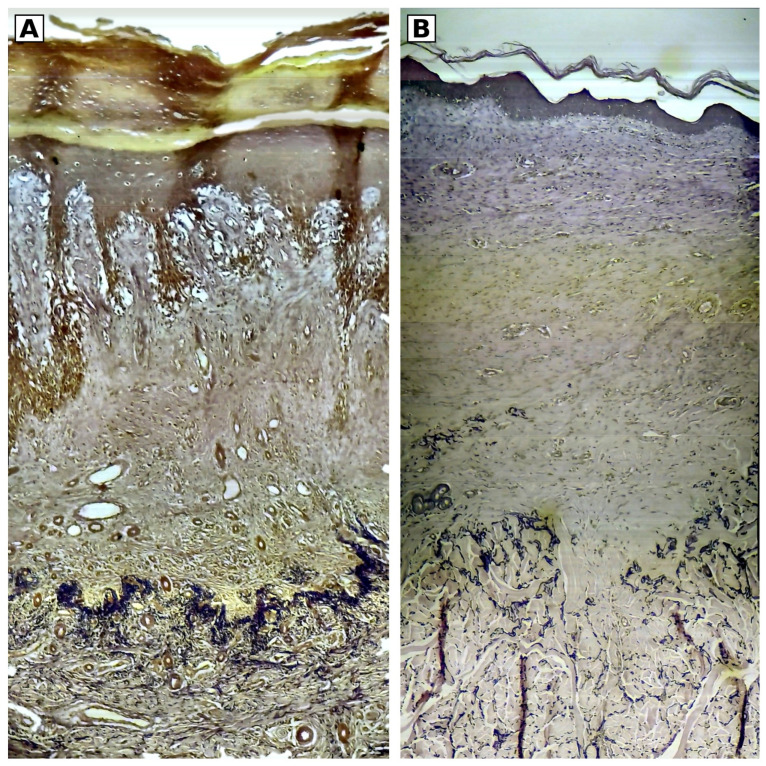
Distinguishing regression from scar by elastic fiber pattern. (**A**) Regression: The papillary dermal elastic fibers are compressed and displaced downward into a shortened “candelabra” layer at the base of the fibrosis, while the deep reticular elastic fibers (lower field) remain normal. (**B**) Scar (prior procedure): The superficial elastic network is lost, with an abrupt transition to coarse reticular fibers and fragmented fiber ends; normal reticular elastic fibers are again visible at the base (lower field). The preserved deep fibers in both panels serve as an internal reference (EVG, ×100). This figure is an original image from the Department of Histopathology, Andreas Syngros Hospital, Athens, Greece, and illustrates previously established histopathological findings.

Kamino et al. demonstrated that melanomas with regression show a distinct compressed layer of thin elastic fibers pushed down from the papillary dermis to the base of the fibrosis, which can be highlighted with an elastinhistostain [5]. This compressed elastic layer preserves a “candelabra” or “fork” pattern, albeit shorter and thicker than normal papillary dermis elastic fibers [5]. In contrast, scars from prior surgical procedures lack this compressed elastic layer and instead show an abrupt transition to thick, coarse, wavy elastic fibers characteristic of reticular dermis, often with cut or fragmented fiber ends [5] (Figure 3). This distinction can be decisive when the history of prior biopsy is uncertain or undocumented [5,10]. Regenerated elastic fibers, which appear short, thin, and haphazardly arrayed, may be seen in both regression and scars, but only scars older than ~3 months show any regenerated fibers at all [5].

### 3.2. Immunophenotype of Regression

The inflammatory infiltrate in regressing melanomas is dominated by CD8+ cytotoxic T cells as the primary effectors of tumor-cell killing, with CD4+ helper T cells also present [11]. Brugés et al. reported that regressing melanomas show higher CD4/CD3 and CD4/CD8 ratios, whereas halo nevi exhibit higher CD8/CD3 ratios, reflecting a more robust cytotoxic response in the benign lesions [11]. Regulatory markers such as FOXP3, PD1, and CD25 are significantly less abundant in regressing melanomas than in halo nevi, suggesting a less active regulatory immune environment in melanoma regression [11]. This pattern contrasts with the strong cytotoxic and regulatory immune reaction seen in halo nevi, which exhibit higher CD8/CD3 ratios and more prominent expression of PD1, FOXP3, and CD25, consistent with an effective immune-mediated destruction of nevus cells [11]. These immunophenotypic differences may aid in distinguishing intensely inflamed melanocytic lesions when conventional morphology is equivocal [11].

#### 3.2.1. Functional Heterogeneity of the Regression Infiltrate

Although immunophenotyping lies outside the strict “old school” morphologic toolkit, the regression infiltrate is functionally heterogeneous and not uniformly anti-tumoral. The observations that follow on infiltrate composition and FOXP3 expression are drawn from an evolving and at times conflicting literature and should be regarded as biologically plausible interpretations rather than established diagnostic facts. The cellular composition of the regression zone governs its net immune polarity—hostile or permissive to residual tumor cells. FOXP3 can be expressed by two biologically distinct cell populations that may coexist within the same regression compartment: melanoma tumor cells and regulatory T cells (Tregs). FOXP3 expression by melanoma tumor cells themselves correlates with greater Breslow depth and higher mitotic index, suggesting that FOXP3-expressing melanoma clones resist immune-mediated destruction and drive tumor progression [12]. Notably, the spatial distribution of FOXP3-positive Tregs is compartmentalized: Tregs preferentially accumulate in non-regressed tumor areas, where they sustain a locally immunosuppressive milieu that favors tumor persistence, whereas regressed areas are relatively Treg-depleted and enriched for dendritic cells [12]. This regional segregation of regulatory and effector populations reinforces the concept that the infiltrate is functionally heterogeneous, with immunosuppression concentrated in the surviving tumor compartment rather than uniformly distributed [13,14,15]. M2-polarized tumor-associated macrophages—identifiable by CD163 immunohistochemistry—may further contribute to immunosuppression and defective angiogenesis within the tumor stroma, contrasting with the anti-tumor activity of M1-polarized macrophages [13,16]. It should be noted, however, that the relationship between regulatory immune cells and regression areas is contested: some studies report that regression-associated lymphocytic areas are relatively Treg-poor and show an absence of local immunosuppression compared with the progressing tumor, allowing a more robust immune response [17]. This distinction helps explain why histologic regression does not reliably confer a favorable prognosis: partial immune activation may clear a proportion of tumor cells while establishing an immunosuppressive niche that permits residual tumor survival.

#### 3.2.2. Tertiary Lymphoid Structures and Maturation-Dependent Immune Polarity

The interpretations in this subsection should be read as mechanistic hypotheses drawn from an evolving literature rather than as established diagnostic facts; the prognostic role of TLS maturation in melanoma regression is an active area of research and has not been validated as a routine diagnostic criterion [18,19,20,21].

**Figure 4:**
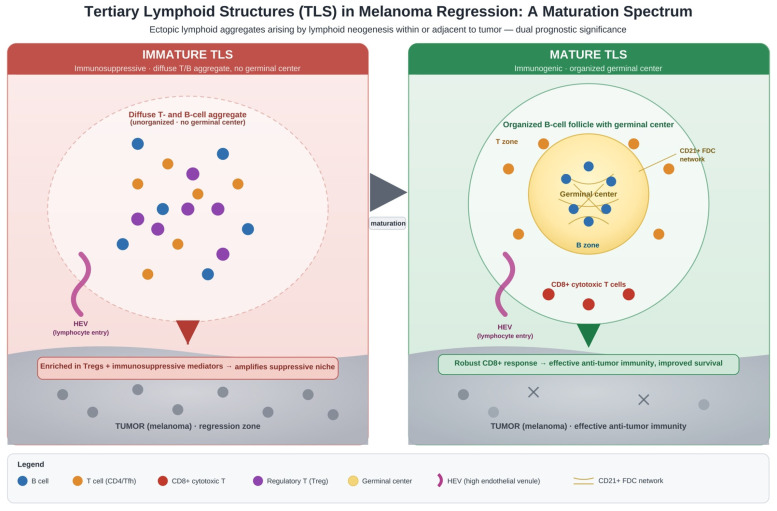
Immature TLSs are recognized by the presence of HEVs and the absence of germinal centers. Mature TLSs are recognizable on H&E by a well-formed germinal center. Immature aggregates may mimic diffuse reactive infiltrate; however, the presence of HEVs and expression of CD21 or CXCL13 by IHC aids confirmation in ambiguous cases.

Tertiary lymphoid structures (TLS)—ectopic lymphoid aggregates arising from lymphoid neogenesis within or adjacent to tumors—can be recognized histologically and range from immature diffuse T and B cell aggregates to mature structures with organized germinal centers, (CD21+) follicular dendritic cell networks, and high endothelial venules (HEV) [18,19,20]. Immature TLS are enriched in regulatory T cells and immunosuppressive mediators, and their presence within the regression zone may amplify rather than counteract the immunosuppressive niche described above [19,20]. In contrast, mature TLS with germinal center formation has been reported to associate with a robust CD8+ cytotoxic response and, in several series, with improved survival in melanoma and other solid tumors, although the strength and consistency of this association continue to be debated [18,19,20,21]. In the context of melanoma regression, the distinction between immature and mature TLS may therefore be prognostically relevant, although this remains to be confirmed in dedicated studies. Mature TLS can be recognized on H&E by a well-formed germinal center; immature TLS lacking germinal centers may be difficult to distinguish from diffuse reactive lymphoid infiltrate on H&E alone—however, the presence of HEVs and CD21 or CXCL13 immunohistochemistry is available for confirmation in ambiguous cases (Figure 4) [19,20]. This maturation spectrum may help explain why histologic regression carries variable and sometimes conflicting prognostic implications: cases with mature peritumoral TLS may represent a genuinely favorable immune response, whereas those with only immature aggregates or diffuse Treg-rich infiltrates may not [19,20,21].

### 3.3. Fibroplasia out of Proportion to Cytology

A practical heuristic is discordance: if melanocytes appear only mildly atypical but the stroma looks highly reactive (fibrotic, inflamed, and/or vascularly altered), suspicion should rise for melanoma, regressed melanoma, or a lesion with an unrecognized invasive component [9]. This “activated” stromal reaction may include lamellar or concentric fibroplasia, increased melanophages, vascular proliferation, and patchy lymphohistiocytic infiltrate, all out of proportion to the apparent degree of melanocytic atypia [9].

### 3.4. Bland Stroma

Most banal acquired nevi show minimal stromal reaction, aside from expected maturation-related dermal remodeling [3]. A dermis that is relatively bland—lacking patchy scar-like fibrosis, abundant melanophages, and irregular inflammation—supports benignity, provided the melanocytic architecture is also reassuring [3]. Traumatized or irritated nevi can show focal fibrosis and inflammation (see Section 7) [3].

### 3.5. Prognostic Implications of Regression

The prognostic significance of regression in primary cutaneous melanoma remains controversial [9]. Some studies have reported that regression is associated with worse prognosis, potentially due to understaging of originally thicker lesions (i.e., the measured Breslow depth reflects only residual melanoma after partial immune-mediated destruction, not the original depth) [9]. Other studies have suggested favorable outcomes (regression as a sign of effective host immune response) or no independent prognostic effect [9]. Inconsistencies in the definition and assessment of regression—including the lack of standardized criteria for evaluating the stage (early/active vs. late), horizontal extent (focal vs. extensive, often using a 75% cutoff in the literature or 50% in MPATH-dx v.2), and depth of regression-associated fibrosis—have contributed to these conflicting findings [9]. As detailed in Section 3.2.2, the maturation state of TLS within the regression zone is an additional explanatory variable, and studies that do not account for it are likely to produce heterogeneous prognostic results [19,20,21]. A universal scheme to objectively define and assess histologic regression—incorporating both the extent and composition of the inflammatory infiltrate, including TLS maturation state—is needed to fully understand its biologic and prognostic significance [9].

## 4. Epidermis, Adnexa, and Inflammation

### 4.1. Epidermal Context

In adult facial skin with severe solar elastosis, a purely junctional lentiginous proliferation should trigger heightened concern for lentigo maligna/melanoma in situ, because this is a favored anatomic-environmental context for melanoma development [3]. In that setting, the absence of an umbrella sign and the presence of severe elastosis under the lesion can reinforce suspicion [3]. Conversely, in a compound or intradermal lesion on sun-damaged skin with a well-developed umbrella sign, the likelihood of long-standing benign nevus increases substantially [3].

### 4.2. Adnexal Preservation vs. Destruction

Benign intradermal/compound nevi often “cuff” adnexa—wrapping around hair follicles and eccrine ducts without disrupting their architecture—and preserve adnexal outlines [3]. Invasive melanoma more often shows infiltrative replacement, distortion, or destruction of adnexal structures, particularly when growth is expansile, desmoplastic, or neurotropic [3]. Adnexal involvement by atypical melanocytes is a well-recognized feature of lentigo maligna/melanoma in situ; extension below the level of the sebaceous duct opening—and particularly into the isthmus and inferior segment down to Adamson’s fringe—is the meaningful criterion, as normal melanocytes regularly populate the upper follicular infundibulum and their presence alone should not be interpreted as malignancy [1,22].

### 4.3. Host Inflammatory Response

Melanomas may show irregular, patchy, sometimes band-like lymphocytic inflammation, often accompanying regression-type changes [3,11]. Interface change and melanophage-rich inflammation can contribute to a pattern that is more suspicious than the sparse, uniform perivascular cuffs typical of many nevi [3,11]. Many nevi show no inflammation or only mild, symmetric perivascular lymphocytes without interface change [3]. Dense, diffuse lymphocytic infiltrates that obscure melanocytes can represent early-stage regression (difficult to distinguish from brisk tumor-infiltrating lymphocytes) or the classical appearance of a halo nevus [11].

## 5. Epidermal Reaction Patterns: Hyperplasia, Pseudoepitheliomatous Hyperplasia, and Effacement

Melanoma provokes a wide spectrum of epidermal responses, ranging from marked hyperplasia (including pseudoepitheliomatous hyperplasia, PEH) to effacement and ulceration [23]. These changes are diagnostically important, may reflect underlying tumor biology, and can be a major source of diagnostic pitfalls, especially on chronically sun-damaged skin where reactive melanocytic hyperplasia is common [23,24].

### 5.1. Terminology and Definitions

Epidermal hyperplasia refers to increased thickness of the viable epidermis (acanthosis), often with overlying hyperkeratosis or parakeratosis [23]. This can be a reactive phenomenon triggered by dermal processes (inflammation and/or neoplasia) or intrinsic epidermal proliferation [23].

Pseudoepitheliomatous hyperplasia (PEH) is a reactive, often irregular, downward proliferation of squamous epithelium characterized by elongated, jagged rete ridges, bulbous epithelial cords extending into the dermis, and squamous eddies or keratin pearls that can closely mimic well-differentiated squamous cell carcinoma [25]. PEH is classically associated with chronic infections (deep fungal, atypical mycobacterial), chronic inflammatory dermatoses, and various benign and malignant neoplasms [25].

Melanocytic hyperplasia denotes an increase in melanocytes confined to the basal layer without formation of true nests [24]. This pattern underlies benign entities such as lentigo simplex, solar lentigo, and the macular background of nevus spilus, and it can also occur as a reactive phenomenon over adnexal neoplasms, scars, or areas of chronic inflammation [24,26]. Melanocytic hyperplasia must be distinguished from lentigo maligna/melanoma in situ, which also features lentiginous melanocytic proliferation but with architectural, cytologic, and contextual features of malignancy [1,22,27,28].

Epidermal effacement/consumption describes thinning or near-loss of the epidermal layers overlying a melanoma, often with attenuation of the rete ridge pattern, diminished keratinocyte layers, and scattered apoptotic or dyskeratotic keratinocytes [29]. Effacement is often adjacent to ulceration or fissuring and may represent a harbinger of impending ulceration [29].

### 5.2. Epidermal Hyperplasia and Melanoma-Driven Angiogenesis

McCarty and colleagues systematically examined primary cutaneous melanomas and their overlying epidermis, correlating epidermal thickness with tumor depth and microvessel density [23,30]. Thin melanomas (Breslow thickness 0.5–1.0 mm) did not show epidermal hyperplasia [23]. In contrast, intermediate and thick melanomas (1.0–10.0 mm Breslow thickness) frequently exhibited epidermal hyperplasia overlying the tumor [23].

Epidermal hyperplasia correlated with decreased interferon-β (IFN-β) expression in keratinocytes directly overlying intermediate/thick melanomas and with increased microvessel density within the melanoma [23]. In xenograft and in vitro work in the same study, only metastatic melanoma cell lines induced epidermal hyperplasia and conditioned media effects on keratinocyte proliferation, supporting a paracrine model linking epidermal hyperplasia to a pro-angiogenic microenvironment [23].

Diagnostic implications: When faced with a melanocytic lesion beneath a markedly hyperplastic epidermis, the differential diagnosis includes melanoma (particularly intermediate/thick invasive melanoma with angiogenesis-related hyperplasia), Spitz nevus, and nevus with reactive/irritation changes; classification requires integration of architecture, cytology, and dermal maturation/mitoses/stromal reaction [1,23].

### 5.3. Pseudoepitheliomatous Hyperplasia (PEH) Associated with Melanoma

PEH can occur over melanocytic lesions and may obscure an underlying melanoma, posing a significant diagnostic trap [25]. Mott et al. reported melanomas with overlying PEH and described SCC-like and seborrheic keratosis-like PEH patterns that can mimic keratinocytic neoplasia [25]. In their cases, melanoma could be partially obscured by PEH, with atypical melanocytes interdigitating between epithelial cords and nests; pigment and junctional/dermal melanocytic atypia were practical clues [25].

EGFR immunohistochemistry in that series showed strong basal keratinocyte staining in hyperplastic and adjacent epithelium, absent or weak staining in melanoma cells, and strong staining in underlying macrophages, arguing against a simple EGFR-driven melanoma cell mechanism for PEH [25]. EGFR immunostaining is not recommended as a routine diagnostic tool in this context.

When faced with exuberant PEH, search deliberately for a melanocytic component before signing out SCC, and liberal use of melanocytic immunostains (e.g., SOX10, Melan-A/MART-1, MITF, S-100) is warranted in ambiguous cases [25].

### 5.4. Epidermal Effacement and Prognostic Associations

**Figure 5:**
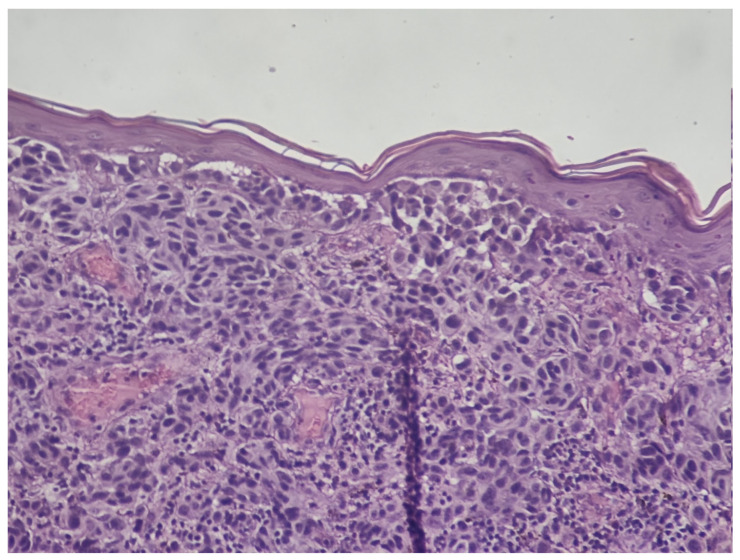
Epidermal effacement overlying melanoma. Tumor gives the impression of “consuming” the epidermis (H&E, ×200). This figure is an original image from the Department of Histopathology, Andreas Syngros Hospital, Athens, Greece, and illustrates previously established histopathological findings.

Epidermal effacement has been reported more frequently in melanoma than in Spitz nevi and may be a useful morphologic clue in spitzoid gray-zone lesions (Figure 5) [29]. Hantschke et al. reported effacement in 53 of 75 melanomas (71%) compared with only 10 of 75 Spitz nevi (13%). In problematic spitzoid lesions with comparative genomic hybridization (CGH), effacement was more frequent in lesions ultimately classified as malignant [29]. Effacement is also associated with ulceration-adjacent change and with other adverse histologic correlates (greater thickness, higher mitotic rate, vertical growth phase, ulceration) in reported cohorts [29,31].

Conversely, paratumoral epidermal hyperplasia in areas adjacent to thick melanoma has been associated with better prognosis in at least one series, suggesting that directionality of epidermal change (hyperplasia vs. effacement) may reflect biologic behavior, though neither feature is incorporated into formal staging [29,30].

Practical utility: Effacement is not specific for melanoma (it can occur in ulcerated/irritated benign lesions), but its presence should prompt heightened scrutiny of melanocytic architecture and cytology [29].

## 6. A Practical Stepwise Diagnostic Framework

The clues discussed above are most useful when integrated into an explicit diagnostic pathway rather than applied in isolation. For a pigmented lesion with epidermal hyperplasia, architectural disturbance, or occurring on sun-damaged skin, the following three-step framework guides the reader from scanning magnification to high-power assessment and, when appropriate, ancillary testing. Table 1 summarizes the main non-melanocytic clues, the direction in which each points, the current level of evidence, and the ancillary test most useful for the corresponding diagnostic uncertainty.

**Table 1:** Summary of non-melanocytic histological clues, the diagnosis they favor, current level of evidence, and the most appropriate ancillary test.

Histological Clue	Favors	Level of Evidence	Suggested Ancillary Test
Umbrella sign (central reduction in elastosis)	Nevus	Single-cohort, qualitatively corroborated [3,6,7,8]	Elastin histostain to confirm pattern [5]
Purple fiber sign (purple-tinged entrapped elastotic fibers)	Nevus	Single-cohort, preliminary [3]	None specific; H&E-dependent [3]
Stromal blending/adnexal cuffing	Nevus	Established morphologic criteria [1]	SOX10/Melan-A to map extent [25]
Displacement/compression of solar elastosis	Melanoma	Established [3,4,5]	Elastin histostain [5]
Regression (compressed elastic layer, melanophages, fibrosis)	Melanoma (context-dependent)	Established morphology; prognostic value variable [5,9]	Elastin histostain or immunostain to separate regression from scar [5]; SOX10 for residual melanocytes [25]
Disproportionate fibrosis/adnexal destruction	Melanoma	Established morphologic criteria [1,29]	p16, Ki-67, FISH/CGH if atypia present [22]
Epidermal effacement (“consumption”)	Melanoma (not specific)	Established but non-specific [29,31]	SOX10/Melan-A [25]

Step 1—Low-power scanning. Assess the following architectural features: symmetry, circumscription, relationship of the proliferation to solar elastosis, epidermal effacement, ulceration, epidermal hyperplasia, and the character of the stromal reaction [3,29].

Step 2—High-power assessment. Distinguish features favoring nevus—the umbrella sign, the purple fiber sign, stromal blending, and adnexal cuffing—from features favoring melanoma, such as compressed or displaced elastosis, regression, disproportionate fibrosis, and adnexal destruction [3,5,9].

Step 3—Ancillary testing. Select ancillary tests according to the specific uncertainty raised at Steps 1 and 2: p16, Ki-67, and SOX10 by immunohistochemistry, and FISH or CGH/array-CGH for borderline lesions where management hinges on clarifying risk [22].

The remainder of this section expands on each step in routine sign-out.

### 6.1. Low Power Assessment (Step 1)

Evaluate symmetry, silhouette, breadth, degree of solar elastosis and any umbrella sign or purple fiber sign in sun-damaged skin (assess the central one-third for umbrella sign, not periphery), and presence of epidermal effacement, ulceration, fissuring, or paratumoral hyperplasia [3,29]. Note the overall architecture (circumscription, pushing vs. infiltrative border) and distribution of any inflammatory infiltrate [3].

### 6.2. Characterize Epidermal Change

Distinguish simple acanthosis from true PEH with irregular epithelial cords, squamous eddies, and SCC-like or seborrheic keratosis-like patterns [25]. Note hyperkeratosis/parakeratosis patterns (psoriasiform, compact orthokeratosis, etc.) and identify areas of effacement, thin residual epidermis, or dyskeratotic keratinocytes [25,29]. If PEH is present, deliberately search for an underlying melanocytic component before diagnosing SCC [25].

### 6.3. Evaluate Melanocytic Component (Step 2)

Assess distribution (lentiginous vs. nested vs. mixed; confluence; adnexal involvement-particularly follicular extension, a key feature of lentigo maligna), pagetoid spread (extent, density, level reached in epidermis), cytology (epithelioid vs. small round vs. spindle; pleomorphism; nucleoli; nuclear-to-cytoplasmic ratio), and dermal component (maturation gradient, mitoses, necrosis, host response [lamellar fibroplasia, inflammatory infiltrate, melanophages]) [1,22].

### 6.4. Evaluate Stromal Features and Elastosis (Step 2)

Look for elastosis patterns: umbrella sign (central reduction favoring nevus), purple fiber sign (high specificity for nevus), or displacement/compression of elastosis (favoring melanoma) [3]. If regression is suspected, look for a compressed elastic layer at the base of fibrosis (the displaced papillary dermal elastic layer described by Kamino et al. [5]), displaced elastosis, melanophages, fibrosis character (lamellar vs. scar-like vs. desmoplastic), and vascular changes [5]. Consider elastin histostain or immunostain if elastosis patterns or regression are ambiguous [5]. Assess overall stroma: bland and inactive (pro-nevus) vs. activated/fibrotic/inflamed out of proportion to melanocytic cytology (red flag for melanoma or regression) [3,9].

### 6.5. Distinguish Key Entities

- Melanoma with overlying simple hyperplasia: Expect dermal invasion, cytologic atypia, lack of maturation, possible mitoses; hyperplastic epidermis usually symmetric and regular (acanthotic but not PEH-like); this may correlate with angiogenesis in thicker melanomas [23].
- Melanoma with PEH: Irregular squamoid cords extend into dermis with SCC-like or SK-like patterns; be vigilant for interspersed atypical melanocytes; use melanocytic immunostains (SOX10, Melan-A, MITF) liberally if the melanocytic component is suspected but obscured [25].
- Watch for benign or dysplastic nevus on sun-damaged skin with umbrella sign and/or purple fiber sign, limited cytologic atypia, and maturation [3].
- Atypical junctional melanocytic hyperplasia/AIMP (Atypical Intraepidermal Melanocytic Nevus) on actinic skin: This includes worrisome features for melanoma in situ but incomplete criteria; discuss as such in the report and manage with excision and margins similar to melanoma in situ (5–10 mm) often [22,32].
- Reactive melanocytic hyperplasia over adnexal tumor, scar, or inflammatory process: This includes limited basal increase, no pagetoid spread, no significant cytologic atypia, and no adnexal extension [24,26].
- Melanoma with regression: Look for compressed elastic layer at base of fibrosis (displaced papillary dermal elastic layer [5]), displaced elastosis, melanophages, lamellar fibrosis, and immunophenotype clues if needed (CD4 predominance and lower regulatory T cell markers compared to halo nevi) [5,9,11].
- Halo nevus (Sutton nevus): Dense lymphocytic infiltrates obscure nevus cells, with a higher CD8/CD3 ratio and greater PD1/FOXP3/CD25 expression compared to regressing melanoma; these are usually symmetric and circumscribed when visible [11].

### 6.6. Ancillary Studies (Step 3)

Use Melan-A/SOX10/MITF to highlight the full extent of melanocytes, particularly under PEH, dense hyperkeratosis, or when regression obscures the junctional component [25]. Apply Ki-67, p16, PRAME (with appropriate caveats regarding specificity) in challenging lesions [22]. Consider FISH/CGH or gene expression profiling in bona fide MELTUMP (Melanocytic Tumor of Uncertain Malignant Potential)/BMT (Borderline Melanocytic Tumor), where management hinges on clarifying risk, or in spitzoid lesions with ambiguous morphology [22]. Elastin histostain can clarify the umbrella sign, compressed elastic layer in regression, and distinction from surgical scar [5]. When non-melanocytic clues do not resolve the differential, and the lesion falls into the gray zone, explicit communication of diagnostic uncertainty using accepted terminology (SAMPUS, MELTUMP) with recommended margins is preferable to a forced binary benign/malignant label [22,32].

## 7. Key Pitfalls

- Thick invasive melanoma can obliterate elastosis centrally and mimic an umbrella-like clearing; check periphery for displacement/compression and integrate overall architectural features (asymmetry, invasion, high-grade cytology) [3].
- Small lentiginous junctional nevi on sun-damaged skin may lack the umbrella sign due to small size or recent development; absence of the umbrella sign is not diagnostic of melanoma in isolation [3].
- Assess the umbrella sign in the central one-third of the lesion, not the periphery, to avoid false-negative interpretation from the shoulder phenomenon [3].
- Purple fiber sign depends on H&E staining characteristics; treat it as a specific supportive clue when present, but its absence does not imply melanoma [3].
- Regression vs. scar: elastin histostain is often decisive—regression shows a compressed layer of thin papillary dermal elastic fibers displaced to the base of fibrosis, whereas scars lack this layer and show an abrupt transition to thick reticular dermal elastic fibers; scars <3 months can lack elastic fibers, and older scars may show regenerated thin, fragmented fibers [5].
- Features like poor circumscription, lentiginous proliferation, occasional suprabasal melanocytes, and mild atypia are not specific for melanoma, particularly on sun-damaged skin or in irritated/recurrent nevi; absence of maturation and true dermal mitotic activity are more specific [33].
- With exuberant PEH (especially SCC-like pattern), search deliberately for a melanocytic component before signing out SCC; liberal use of melanocytic immunostains is warranted [25].
- Epidermal effacement is more common in melanoma than in nevi but is not specific; interpret it in full context [29].
- Early-stage regression (dense lymphocytic infiltrate obscuring melanocytes) overlaps with brisk TILs and is subjective; many pathologists emphasize late-stage regression features for reproducibility [9].

## 8. Discussion

Non-melanocytic clues describe lesion-microenvironment interactions not fully captured by melanocyte cytology, immunohistochemistry, or any single ancillary assay [3]. Quantitative support for elastosis-based signs-umbrella sign with high sensitivity for nevus in Wood and Harvey’s sun-damaged skin cohort; purple fiber sign with very high specificity for nevus-reinforces the continuing value of contextual assessment in sun-damaged skin [3]. Elastic fiber patterns in regression (compressed “candelabra” layer at base of fibrosis) can distinguish true regression from surgical scar (abrupt transition to thick reticular fibers), a distinction that may be difficult or impossible on H&E alone [5].

Epidermal reaction patterns (hyperplasia linked to angiogenesis in thick melanomas; PEH that can obscure melanoma; effacement as a red flag in spitzoid lesions) can both obscure melanoma and provide diagnostic warnings, while stromal regression-type changes, immunophenotyping of inflammation, and elastosis displacement remain practical safeguards against both over- and under-diagnosis [3,5,9,11,23,25,29]. Ancillary tools (e.g., PRAME, Ki-67, p16; FISH/CGH; and gene expression profiling) are best used to resolve a differential diagnosis already framed by morphology [22]. They are helpful adjuncts but not replacements for careful pattern analysis [22,32]. In daily sign-out, the fastest and often most informative step remains the “old school” low-power scan integrating the non-melanocytic clues cataloged in the stepwise framework of Section 6 [3,5].

The quantitative data supporting the umbrella and purple fiber signs derive from a single cohort of 81 actinically damaged lesions [3]; independent prospective validation in larger, multicenter series is needed before these thresholds are adopted as formal diagnostic criteria. The qualitative umbrella sign concept is, however, independently corroborated in a separate practical review of melanocytic diagnosis, in a standard dermatopathology textbook, and in a recent peer-reviewed update on lentigo maligna, all of which support the underlying shielding principle even as the precise predictive values remain single-cohort [6,7,8].

A further limitation concerns interobserver reproducibility. Because the focus of this review is subtle, often low-contrast histological clues, their practical value depends on whether different pathologists can recognize them consistently. To date, no formal interobserver concordance study has been published for the umbrella sign or the purple fiber sign specifically; establishing kappa-level agreement for these signs across pathologists of differing experience is an important and currently unmet research need. This should be regarded as an open gap. This uncertainty must be read against the broader and well-documented problem of interobserver variability in melanocytic pathology generally, which is greatest for borderline lesions. Subtle elastotic and stromal clues are therefore likely to be most reliably assessed by pathologists with dedicated dermatopathology experience, and elastin immunohistochemistry may improve consistency by rendering elastosis patterns more objective than H&E alone [5]. General surgical pathologists without subspecialty dermatopathology training may find these low-contrast clues harder to apply reproducibly, and in that setting, the signs are best treated as prompts to seek expert review or ancillary confirmation rather than as independent decision points. Pending dedicated concordance studies, these signs are best used as supportive features within a multifactorial assessment rather than as decisive, stand-alone criteria [3,33].

On chronically sun-damaged skin, the “old school” low-power scan remains essential because it is the framework within which modern adjuncts are most productive [3,34]. Ancillary molecular and immunohistochemical tests are most productive when the differential has already been framed by careful morphologic assessment of the melanocyte–epidermis–stroma interaction [22]. Atypical melanocytic proliferations inhabit a genuine gray zone; for these, clear communication of uncertainty and risk-adapted management is often more valuable than an artificially definitive binary label [22,32]. In answer to the question posed in the title: these non-melanocytic clues remain indispensable—not as replacements for cytology, immunohistochemistry, or molecular testing, but as the morphologic scaffold that makes those adjuncts most effective.

## 9. Conclusions

Non-melanocytic histopathological features—the pattern of dermal solar elastosis, the character of the stromal reaction, regression and its associated tertiary lymphoid structures, and the behavior of adjacent adnexal structures—provide valuable ancillary clues that can support the distinction between benign melanocytic proliferations and melanoma, particularly in sun-damaged skin. Signs such as the umbrella and purple fiber signs are promising but remain preliminary observations derived from limited cohorts, and should be applied as supportive prompts rather than as independent diagnostic criteria. Their reproducibility across observers of differing experience has not yet been formally established and represents an important research need. Used within the stepwise framework proposed here—low-power architectural assessment, high-power evaluation of competing benign and malignant clues, and the judicious deployment of ancillary tests—these non-melanocytic features can meaningfully strengthen diagnostic confidence at the bench while awaiting validation in larger, prospective studies.

## Data Availability

No new data were created or analyzed in this study. Figure 1, Figure 2, Figure 3 and Figure 5 are original, fully anonymized archival histological teaching images from the Department of Histopathology, Andreas Syngros Hospital, Athens, Greece, used to illustrate previously established histopathological concepts; Figure 4 is an original schematic diagram prepared by the author. Data sharing is not applicable to this article.
